# Growth hormone treatment does not augment the anti‐diabetic effects of liraglutide in UCD‐T2DM rats

**DOI:** 10.1002/edm2.392

**Published:** 2022-12-08

**Authors:** Michael M. Swarbrick, Chad L. Cox, James L. Graham, Lotte B. Knudsen, Kimber Stanhope, Kirsten Raun, Peter J. Havel

**Affiliations:** ^1^ Departments of Nutrition and Department of Molecular Biosciences, School of Veterinary Medicine University of California, Davis One Shielad Avenue Davis California USA; ^2^ Present address: Bone Research Program, ANZAC Research Institute The University of Sydney Concord New South Wales Australia; ^3^ Present address: Concord Clinical School, Faculty of Medicine and Health The University of Sydney Australia; ^4^ Present address: Novo Nordisk A/S Maaloev Denmark

**Keywords:** diabetes mellitus, food intake, growth hormone, insulin, liraglutide, obesity

## Abstract

**Introduction:**

The incretin hormone glucagon‐like peptide‐1 (GLP‐1) slows gastric emptying, increases satiety and enhances insulin secretion. GLP‐1 receptor agonists, such as liraglutide, are used therapeutically in humans to improve glycaemic control and delay the onset of type 2 diabetes mellitus (T2DM). In UCD‐T2DM rats, a model of polygenic obesity and insulin resistance, we have previously reported that daily liraglutide administration delayed diabetes onset by >4 months. Growth hormone (GH) may exert anti‐diabetic effects, including increasing β‐cell mass and insulin secretion, while disrupting GH signalling in mice reduces both the size and number of pancreatic islets. We therefore hypothesized that GH supplementation would augment liraglutide's anti‐diabetic effects.

**Methods:**

Male UCD‐T2DM rats were treated daily with GH (0.3 mg/kg) and/or liraglutide (0.2 mg/kg) from 2 months of age. Control (vehicle) and food‐restricted (with food intake matched to liraglutide‐treated rats) groups were also studied. The effects of treatment on diabetes onset and weight gain were assessed, as well as measures of glucose tolerance, lipids and islet morphology.

**Results:**

Liraglutide treatment significantly reduced food intake and body weight and improved glucose tolerance and insulin sensitivity, relative to controls. After 4.5 months, none of the liraglutide‐treated rats had developed T2DM (overall *p* = .019). Liraglutide‐treated rats also displayed lower fasting triglyceride (TG) concentrations and lower hepatic TG content, compared to control rats. Islet morphology was improved in liraglutide‐treated rats, with significantly increased pancreatic insulin content (*p* < .05 vs. controls). Although GH treatment tended to increase body weight (and gastrocnemius muscle weight), there were no obvious effects on diabetes onset or other diabetes‐related outcomes.

**Conclusion:**

GH supplementation did not augment the anti‐diabetic effects of liraglutide.

## Graphical abstract

Growth hormone (GH) may directly exert anti‐diabetic effects on pancreatic islets, increasing beta‐cell mass and insulin secretion. Here we used a rodent model of type 2 diabetes (UCD‐T2DM rats) to investigate whether GH supplementation could augment the anti‐diabetic effects of liraglutide (a GLP‐1 receptor agonist) treatment.

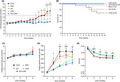

## 1 INTRODUCTION

More than 400 million people worldwide are currently afflicted with type 2 diabetes (T2DM),^1^ a disease characterized by insulin resistance and inadequate ß‐cell/islet compensation, which leads to chronic hyperglycemia. Common complications from T2DM include hypertension, hyperlipidaemia, chronic kidney disease, cardiovascular disease, blindness, nephropathy and amputations.^2^ Insulin resistance is the initial defect in T2DM,^3^ and is defined as impaired insulin‐stimulated glucose uptake and/or suppression of adipose tissue lipolysis. Studies in humans and animal models have established that in susceptible individuals, insulin resistance and T2DM are precipitated by ectopic accumulation of fatty acids in non‐adipose tissues (e.g., liver and muscle). In these tissues, excess fatty acids enter toxic metabolic pathways that can promote apoptosis (lipotoxicity).^4^ Dysregulation of adipose tissue lipolysis, particularly in visceral adipose tissue, may further contribute to the development of insulin resistance, via increased flux of free fatty acids (FFAs) to the liver via the portal vein.^5^ Altered secretion of adipose‐derived hormones (adipokines) and inflammatory mediators can also exacerbate systemic insulin resistance.^6^

In recent years, the rapid rise in the prevalence of T2DM has necessitated the development of new approaches for treatment and prevention, including those based upon gut‐derived hormones that modulate pancreatic hormone secretion (“incretins”).^7^ Incretin hormones, such as glucagon‐like peptide‐1 (GLP‐1) and glucose‐dependent insulinotropic polypeptide (GIP) potentiate insulin secretion, increase satiety and slow gastric emptying. Incretin‐based T2DM therapies, including GLP‐1 receptor agonists and dipeptidyl peptidase 4 inhibitors, are generally well tolerated and have been used for many years to regulate glucose metabolism and manage T2DM in humans.^8^, ^9^ Recent studies in humans suggest that in addition to its effects on food intake, liraglutide also suppresses adipose tissue lipolysis and reduces hepatic lipogenesis, concomitantly improving measures of liver function.^10^

Growth hormone (GH) is known to counteract many of insulin's metabolic effects, increasing hepatic gluconeogenesis and stimulating lipolysis, for example (reviewed in^11^). At the same time, GH exerts beneficial effects on pancreatic islets, including increasing β‐cell proliferation, glucose‐stimulated insulin secretion (GSIS), and the expression and biosynthesis of insulin.^12^ Interestingly, disruption of GH signalling in growth hormone receptor (GHR)‐deficient mice leads to reduced β‐cell mass (due to decreased islet replication and growth), impaired glucose tolerance and lower pancreatic insulin content, compared with wild‐type mice.^13^ Although these findings in GHR‐deficient mice were confounded by dwarfism and increased adiposity, more recent studies of mice lacking GHR specifically in β‐cells demonstrated a blunted first‐phase GSIS and reduced β‐cell hyperplasia when mice were challenged with a high‐fat diet.^14^ Taken together, these findings suggest that GH and its receptor are required for the appropriate insulinotropic adaptation (β‐cell hyperplasia and increased compensatory insulin secretion) during the development of obesity and insulin resistance.

We have developed a rat model of polygenic T2DM with adult‐onset obesity and insulin resistance, which faithfully recapitulates many of the features of adult‐onset T2DM in humans.^15^ Male UCD‐T2M rats develop progressive hyperglycemia, due to inadequate β‐cell compensation for insulin resistance, which leads to overt diabetes at ~4 to 6 months of age. We have previously demonstrated that chronic administration of liraglutide (0.2 mg/kg twice daily) delayed diabetes onset in UCD‐T2DM rats by ~4 months compared with vehicle‐treated ad libitum‐fed controls, and by ~1.3 months compared with vehicle‐treated rats matched for food intake and body weight.^16^ While inhibition of food intake (leading to reduced adiposity) was likely an important mechanism contributing to the anti‐diabetic effects of liraglutide, we hypothesized that supplementing liraglutide with GH in this model would augment the anti‐diabetic effects of liraglutide by preserving pancreatic islet integrity/health and ß‐cell function.

## 2 MATERIALS AND METHODS

### 2.1 Animals, housing, and treatment

UCD/T2DM rats (*Rattus norvegicus domestica*) used for this study were obtained from our established breeding colony. These rats were bred by crossing obese Sprague–Dawley rats (with insulin resistance resulting from polygenic adult‐onset obesity) with Zucker diabetic fatty‐lean rats (possessing a defect in pancreatic beta‐cell function but normal leptin signalling).^15^ Male rats were singly housed in polycarbonate cages throughout the treatment period, and the facility was maintained at a constant 22°C with a 14 h light/10 h dark cycle. Drinking water and ground diet (Rat Diets 5012, Lab Supply) were provided ad libitum, and the diet was provided in spill‐resistant containers.

From 2 to 6.5 months of age, rats were assigned to one of the following weight‐matched experimental groups (control, CON; food‐restricted, RES; liraglutide, LIRA; growth hormone, GH; growth hormone + liraglutide, GH + LIRA; *n* = 16/group), using a random‐number generator in Microsoft Excel. Based on results from our previous liraglutide study,^16^ this sample size gave us 85% power to detect a difference in diabetes prevalence (our primary outcome variable) between RES and LIRA rats, at a two‐sided significance of 0.01 (to allow for multiple comparisons). No rats were excluded, and potential confounders (such as treatment order or cage location) were not controlled for in the study.

Liraglutide (0.2 mg/kg, twice daily, morning and evening, provided by Novo Nordisk A/S, Denmark) and GH (0.3 mg/kg once daily, morning, provided by Bresatec, Adelaide, South Australia) were administered subcutaneously, and CON and RES rats received equivalent volume injections of vehicle. Body weight and food intake were measured 3 times per week. Food intake in RES rats was restricted, so that their weight gain was matched to LIRA and GH + LIRA rats. Non‐fasting blood glucose concentrations were measured weekly, with a OneTouch Ultra Glucometer (LifeScan, Inc). The presence of overt diabetes was defined by two sequential blood glucose readings >200 mg/dl (11.11 mmoL/L).^15^ The experimental protocols were approved by the UC Davis Institutional Animal Care and Use Committee.

At the end of the treatment period, rats were anaesthetised with an overdose of pentobarbital (200 mg/kg, *i.p*.). The pancreas was dissected out, cleaned and weighed. Pancreas samples were placed in 4% paraformaldehyde (v/v) for quantification of islets, and a systematically defined portion was placed in acid/ethanol for determination of insulin and glucagon content (see below). The liver, gastrocnemius muscle and the epididymal, mesenteric, retroperitoneal and subcutaneous adipose tissues were dissected out, weighed and frozen in liquid nitrogen for later analysis.

### 2.2 Energy expenditure

At 3 months of age, 24‐h energy expenditure was measured using a rodent metabolic monitoring system (AccuScan Instruments). Rats were acclimated to the chambers for 8 hours immediately prior to the 24‐h data collection period, and they received their normal diet and water ad libitum. Food intake was also monitored. Data collection began following the morning injection of liraglutide, GH or vehicle and was paused for the evening injection. Energy expenditure was calculated using the equation (3.941 × VO_2_) + (1.106 × VCO_2_), and as expressed as kcal/minute and normalized by body weight.

### 2.3 Blood collection and biochemical measurements

Fasting blood samples (1.0 ml) were obtained every 4 weeks. Samples were collected from the tail into tubes containing EDTA, and plasma was separated by centrifugation for measurement of TG, total cholesterol, FFA, glucose, insulin and leptin concentrations. TG, total cholesterol and glucose concentrations were measured spectrophotometrically using enzymatic reagents from Fisher Diagnostics. FFA concentrations were measured with an enzymatic colorimetric assay from Wako Chemicals USA, Inc. Insulin and total GLP‐1 were measured by electrochemiluminescence (Meso Scale Discovery). Leptin and adiponectin concentrations were measured by RIA (Millpore Sigma).

### 2.4 Oral glucose tolerance tests (OGTT)

After 1.5 months of treatment (3.5 months of age), glucose tolerance, GSIS and glucose‐stimulated GLP‐1 secretion were assessed during an OGTT. Rats were fasted overnight and then received a 50% dextrose solution (1 g/kg) by oral gavage. Blood was collected from the tail at 0, 5, 15, 30, 45, 60, 90 and 120 minutes after glucose administration for analysis of plasma glucose insulin and total GLP‐1 concentrations.

### 2.5 Pancreatic insulin and glucagon content and immunohistochemistry

Pancreata were collected and insulin and glucagon content were determined as previously described.^17^ Weighed samples of clean, blotted pancreas were minced and sonicated in standard volume of acid/ethanol. After overnight extraction, the samples were centrifuged, supernatants were removed and assayed for insulin and glucagon by radioimmunoassay (Millpore Sigma).

The remaining pancreas was fixed in 4% paraformaldehyde for 48 h at 4°C and embedded in paraffin. Tissues were then cut into 3 μm thick sections, deparaffinized in a xylene ethanol series and placed in Tris‐EDTA buffer for antigen retrieval (10 mM Tris, 1 mM EDTA, 0.05% Tween, pH 9.0). Sections were then blocked in 5% bovine serum albumin and immunostained for insulin and glucagon using a monoclonal anti‐mouse antibody (1:100) and a monoclonal anti‐rabbit antibody (1:50), respectively (both antibodies were obtained from Santa Cruz Biotechnology). Detection of the primary antibodies was performed using Alexa Flour 488 anti‐goat and Alexa Flour 633 anti‐mouse secondary antibodies (1:200, Invitrogen). Nuclei were detected using 4′,6′‐diamino‐2‐phenyl inodole (DAPI), included in the mounting solution (Invitrogen). Ten sections taken from each pancreas and slides from *n* = 4 animals per group were imaged for quantification. Total islet area (derived from *n* = 27–89 islets per slide) was determined using Image J software.^18^

### 2.6 Liver and Muscle TG Content

TG content in liver and muscle tissue were determined by the Folch method.^19^ Weighed tissue samples were homogenized in 2:1 methanol: chloroform. After overnight extraction, 0.7% sodium chloride was added. The aqueous layer was aspirated, and duplicate aliquots of the chloroform/lipid layer were evaporated under nitrogen gas. The lipid was reconstituted in isopropyl alcohol and assayed for TG spectrophotometrically using enzymatic reagents from Thermo DMA.

### 2.7 Statistical analysis

Statistical analysis was performed using GraphPrism software (v9 for macOS). Continuous variables were first assessed for normality using the Kolmogorov–Smirnov test. Comparisons between groups were made by one‐ or two‐way ANOVA (as appropriate) with Dunnett's (or Tukey's) correction for multiple comparisons. Diabetes incidence between groups was assessed with a log‐rank survival test. The quantitative insulin sensitivity check index (QUICKI) was calculated as 1/[log(fasting insulin in ng/ml) + log(fasting glucose in mmol/l)].^20^ In all cases, a two‐sided *p*‐value < .05 was considered statistically significant.

## 3 RESULTS

### 3.1 Liraglutide treatment, with or without GH, reduced food intake, body weight, and adiposity without affecting energy expenditure

**FIGURE 1:**
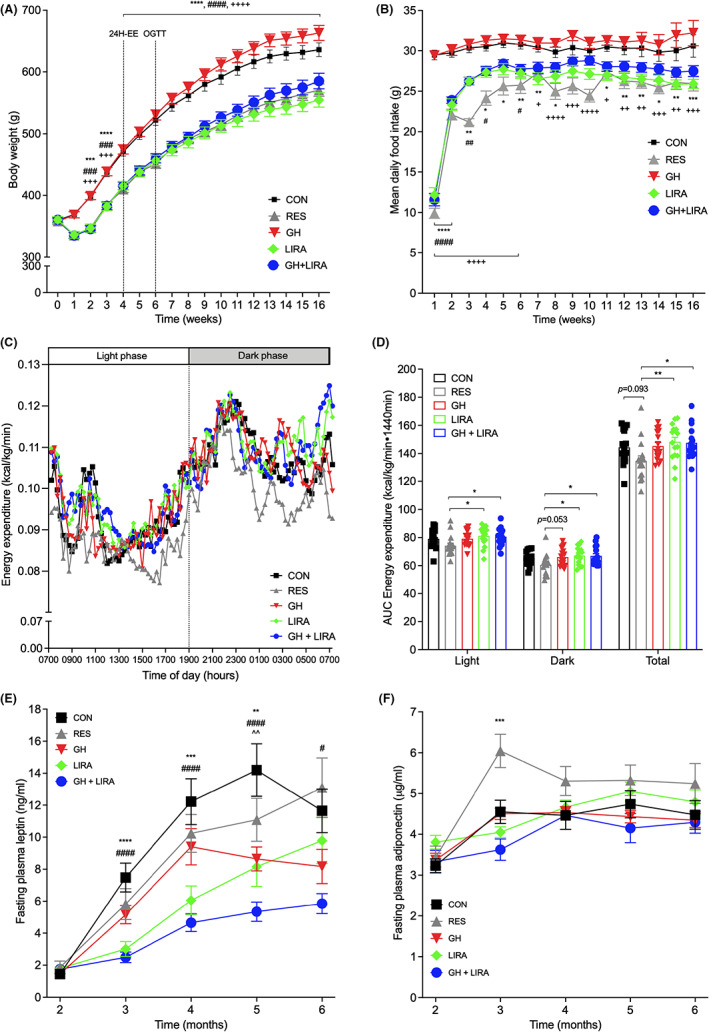
Liraglutide treatment, with or without GH supplementation, reduced food intake, body weight and adiposity in UCD‐T2DM rats. All data are shown as mean ± SEM. 24H‐EE, 24‐h energy expenditure; CON, control; GH, growth hormone; GH + LIRA, growth hormone + liraglutide; LIRA, liraglutide; OGTT, oral glucose tolerance test; RES, food‐restricted. In all cases, *n* = 16/group, *p < .05, ** < .01, ***p < .001 and ****p < .0001 for CON versus LIRA; ^#^ *p* < .05, ^##^ *p* < .01, ^###^ *p* < .001 and ^####^ *p* < .0001 for CON versus GH + LIRA; ^+++^ *p* < .001 and ^++++^ *p* < .0001 for CON versus RES; ^^^^ *p* < .01 for GH versus CON. (A) Body weight during the study. Timepoints for the 24‐EE and glucose tolerance (OGTT) measurements are indicated. (B) Food intake. (C) Energy expenditure. (D) Energy expenditure AUC. (E) Fasting plasma leptin concentrations. (F) Fasting plasma adiponectin concentrations. Differences between groups in (A) and (B) were analysed using two‐way repeated measures ANOVA, with Tukey's tests for post‐hoc comparisons. Differences between groups in (E) and (F) were assessed using one‐way ANOVA at each timepoint, with *p*‐values corrected using Dunnett's multiple comparisons test.

During the first week of treatment, the five groups of rats separated into two main groups: CON and GH rats increased their body weight, while body weight in RES, LIRA, and GH + LIRA rats was reduced by ~9% (*p* = NS, Figure 1A) and maintained a lower trajectory for the remainder of the study. From week 2 onwards, body weights of RES, LIRA GH + LIRA rats were all significantly lower than those of CON rats (*p* < .001 for all comparisons). At the end of the study, mean body weights in RES, LIRA and GH + LIRA rats were 90%, 87% and 92% of CON rats, respectively (*p* < .0001 for all comparisons). Mean body weight in GH‐treated rats tended to be ~4% higher than that of CON rats (662 ± 12 g vs. 636 ± 11 g [mean ± SEM], respectively); however, the difference was not statistically significant.

Treatment with LIRA or GH + LIRA significantly reduced food intake over the course of the study. During the first week, food intake in LIRA and GH + LIRA rats was reduced by 59% and 61%, respectively, compared with CON rats (*p* < .0001, Figure 1B). From week 4 onwards, food intake in LIR and GH + LIRA rats had stabilized at ~90% of that observed in CON rats; and in RES rats, food intake was matched to that observed in LIRA and GH + LIRA rats.

Energy expenditure was assessed after one month of treatment. Energy expenditure in LIRA rats and GH + LIRA rats was, on average, 102.7% and 102.2% of CON rats; however, these differences between groups were not statistically significant (Figures 1C,D). In RES rats, energy expenditure was, on average, 9.3% lower than in CON rats (*p* = .093). Interestingly, 24‐hour energy expenditure was significantly lower in RES rats when they were compared with the liraglutide‐treated groups (*p* < .05 for each comparison), indicating that LIRA prevented the decline in energy expenditure that normally occurs in response to food restriction. There were no significant differences between RES and LIRA/GH + LIRA rats when energy expenditure was analysed according to body weight using linear regression (Figure S1). Instead, the indistinguishable mean body weights and energy intake of RES and LIRA rats suggested that liraglutide‐induced weight loss was predominantly due to reduced food intake. GH treatment did not have any additional effects on these parameters.

Consistent with the differences in body weight and food intake between groups, liraglutide treatment also reduced adiposity (Figure 1E and Table 1). During the treatment period, fasting plasma leptin concentrations were used as an index of adiposity^21^: leptin levels were consistently lower in LIRA and GH + LIRA rats, compared with CON rats (*p* < .001 at 3, 4, and 5 months of age, Figure 1E). Leptin levels were also transiently suppressed in GH‐treated rats, being significantly lower than CON rats at the 5‐month timepoint (*p* < .01). At 6 months of age, only GH + LIRA rats displayed lower fasting plasma leptin concentrations than CON rats (*p* < .05). We also measured concentrations of adiponectin, an insulin‐sensitizing adipokine^22^: a transient elevation in adiponectin concentrations was observed in RES rats (*p* < .001), at one month of treatment (Figure 1F).

**TABLE 1:** Masses of skeletal muscle, liver, and adipose tissue depots at sacrifice.

Variable	CON	RES	GH	LIRA	GH + LIRA
Tissue mass (g)					
Gastrocnemius muscle	3.17 ± 0.06	2.92 ± 0.05**	3.35 ± 0.06	2.85 ± 0.05***	3.09 ± 0.05
Liver	19.6 ± 0.5	16.5 ± 0.5***	19.5 ± 0.6	15.2 ± 0.5***	14.7 ± 0.5***
Epididymal fat	8.8 ± 0.4	7.8 ± 0.5	9.3 ± 0.5	6.3 ± 0.4***	6.7 ± 0.4**
Retroperitoneal fat	10.0 ± 0.4	9.6 ± 0.6	9.8 ± 0.5	7.6 ± 0.6**	7.2 ± 0.5**
Subcutaneous fat	46 ± 2	39 ± 3	50 ± 3	37 ± 3	35 ± 3*
Mesenteric fat	8.3 ± 0.4	7.3 ± 0.5	8.2 ± 0.5	5.8 ± 0.4**	6.0 ± 0.4**
Total adipose tissue	73 ± 3	63 ± 4	77 ± 4	57 ± 4*	55 ± 3**

*Note*: All values are shown as mean ± SEM. Differences between groups were assessed by one‐way ANOVA, with Dunnett's correction for multiple hypothesis testing. All *p*‐values are expressed relative to CON rats: * *p* < 0.05, **p < 0.01, ***p < 0.001. n = 16/group.

Abbreviations: CON, control; GH, growth hormone; GH + LIRA, growth hormone + liraglutide; LIRA, liraglutide; RES, food‐restricted.

At sacrifice, both groups of liraglutide‐treated rats possessed significantly smaller adipose depot weights compared with CON rats (Table 1). Mesenteric, retroperitoneal and epididymal adipose depots from LIRA and GH‐LIRA rats were all significantly smaller than those of CON rats (fat mass was 71%–76% of CON rats, depending on depot, *p* < .01 for all comparisons). Subcutaneous adipose tissue weights were 19% and 24% smaller in LIRA and GH + LIRA rats (*p* = .070 and *p* < .05, respectively), compared to CON rats. Interestingly, none of the adipose depots in RES or GH‐treated rats were significantly smaller than those from CON rats (4%–15% smaller, depending on depot, *p* > .05). Therefore, in addition to reducing food intake and body weight, liraglutide treatment reduced adiposity more than would be expected based on the differences in food intake, and restriction of food intake on its own did not significantly reduce adiposity in UCD‐T2DM rats. There were no clear effects of GH treatment on adiposity.

Masses of gastrocnemius muscle and liver were also measured at sacrifice (Table 1). RES and LIRA rats had significantly smaller gastrocnemius muscles than CON rats (8% and 10% lower than CON rats, respectively, *p* < .01 and *p* < .001). In GH‐treated rats, these muscles were, on average, ~6% larger compared with CON rats (*p* = .069). Liver weights were significantly reduced in RES, LIRA and GH + LIRA rats, compared with CON rats (*p* < .01 for all comparisons, Table 1).

### 3.2 Liraglutide treatment, with or without GH, prevented the development of diabetes in UCD‐T2DM rats and ameliorated insulin resistance

**FIGURE 2:**
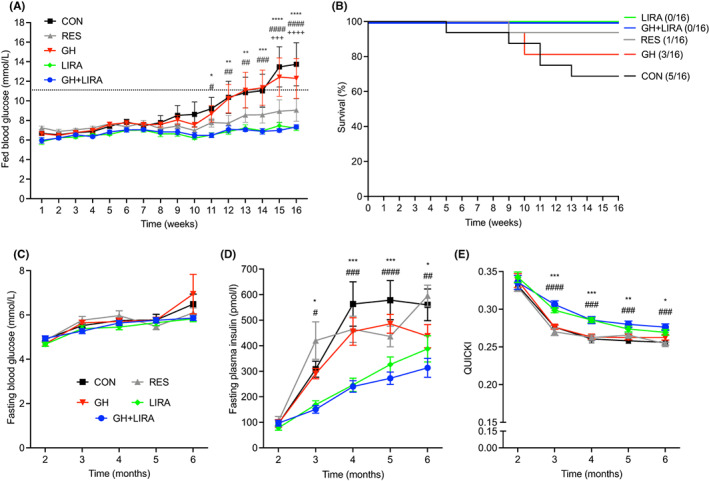
Liraglutide treatment, with or without GH supplementation, completely prevented the development of diabetes in UCD‐T2DM rats. All data are shown as mean ± SEM. CON, control; GH, growth hormone; GH + LIRA, growth hormone + liraglutide; LIRA, liraglutide; RES, food‐restricted; *n* = 16/group. (A) Fed blood glucose concentrations. Differences between groups were analysed using two‐way repeated measures ANOVA, with Tukey's tests for post‐hoc comparisons. The dotted line shows the threshold for overt diabetes, which was defined here as two sequential fed blood glucose readings >200 mg/dl (11.11 mmoL/L). Overall, the interaction between treatment and time was highly significant: *F*_(60, 1125)_ = 2.940, *p* < .0001. Symbols indicate the following significant comparisons: *p < .05, **p < .01, ***p < .001 and ****p < .0001 for CON versus LIRA; ^#^ *p* < .05, ^##^ *p* < .01, ^###^ *p* < .001 and ^####^ *p* < .0001 for CON versus GH + LIRA; ^+++^ *p* < .001 and ^++++^ *p* < .0001 for CON versus RES. (B) Age of diabetes onset (as defined above) in the five experimental groups. The difference in diabetes onset between the groups was significant (log‐rank test, *χ* ^2^ = 11.97, df = 4, *p* = .0191). (C) Fasting blood glucose concentrations. (D). Fasting plasma insulin concentrations. (E) QUICKI assessment of insulin resistance.

During the study, non‐fasting blood glucose concentrations progressively increased in CON rats, from the eighth week onwards (Figure 2A). Similar increases were observed in the GH group, while non‐fasting blood glucose concentrations remained low in the RES, LIRA and GH + LIRA groups. Significant differences between groups were observed from 11 weeks of treatment onwards (CON: 9.2 ± 1.1 mmoL/L [mean ± SEM], LIRA: 6.5 ± 0.2 mmoL/L and GH + LIRA: 6.5 ± 0.2 mmoL/L, *p* < .05 for comparisons). By week 15, a significant reduction of non‐fasting blood glucose concentrations was apparent in the RES group, compared with the CON group (CON: 13.5 ± 2.0 mmoL/L vs. 8.9 ± 1.2 mmoL/L, *p* < .001).

Liraglutide significantly attenuated the onset of overt diabetes; none of the LIRA and GH + LIRA rats developed diabetes during the treatment period, compared with a rate of 5/16 (31%) in CON rats (Figure 2B). Three GH‐treated rats and one RES rat had developed diabetes after 26 weeks. Overall, the differences between treatment groups were significant (log‐rank survival curve, *χ* ^2^ = 11.7, overall *p* = .019).

Despite these differences in diabetes incidence, fasting blood glucose concentrations were not significantly different between the five groups of rats at 2, 3, 4, 5 or 6 months (Figure 2C). Consistent with the development of insulin resistance, fasting insulinemia increased throughout the study in CON, RES and GH‐treated rats, but not in LIRA and GH + LIRA rats (Figure 2D). We also calculated QUICKI, an index of insulin resistance which has been validated against the hyperinsulinaemic‐euglycaemic clamp in rats.^23^ As suggested by the differences in fasting insulin concentrations, LIRA and GH + LIRA rats were protected against the development of insulin resistance (Figure 2E). Neither dietary restriction nor GH treatment protected against insulin resistance in this model.

### 3.3 Liraglutide‐treated rats had improved glucose tolerance, lower fasting insulinemia and reduced GLP‐1 excursions

**FIGURE 3:**
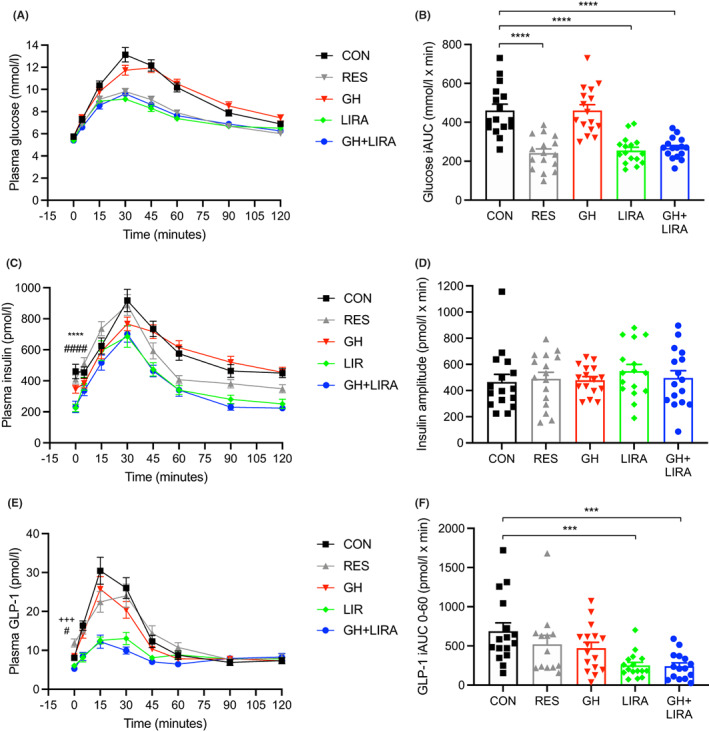
Liraglutide treatment, with or without GH supplementation, improved glucose tolerance, lowered fasting insulin concentrations and reduced GLP‐1 secretion during an OGTT. CON, control; GH, growth hormone; GH + LIRA, growth hormone + liraglutide; LIRA, liraglutide; RES, food‐restricted; *n* = 16/group. Results in (A) (C) and (E) are shown as mean ± SEM, while (B), (D) and (F) are shown as scatterplots with the mean ± SEM shown in columns. Differences between groups were assessed using one‐way ANOVA, with *p*‐values corrected using Dunnett's multiple comparisons test. (A) Blood glucose concentrations during an OGTT. (B) Blood glucose incremental AUC (iAUC) during an OGTT, ****p < .0001 versus CON rats. (C) Plasma insulin concentrations during the OGTT, ****p < .0001 for LIRA versus CON rats, ^####^ *p* < .0001 for GH + LIRA versus CON rats. (D) Amplitude of insulin secretion (pmol/l) for the groups in (C). (E) Plasma concentrations of GLP‐1 during the OGTT, ^#^ *p* < .05 for GH + LIRA versus CON rats; ^+++^ *p* < .001 for RES versus CON rats. (F) GLP‐1 excursions during the OGTT (0–60 min) in individual rats, ***p < .001 versus CON rats.

Glucose tolerance was assessed by OGTT after 1.5 months of treatment (Figure 3). As we found previously, fasting blood glucose concentrations were not significantly different between the five groups of rats (Figure 3A); however, postprandial glucose excursions were significantly lower in both groups of liraglutide‐treated rats, being 45% lower in LIRA rats (iAUC for CON: 460.4 ± 32.3 [mean ± SEM] vs. LIRA: 254.6 ± 17.2 mmoL/L × min, *p* < .0001, Figure 3B) and 42% lower in GH + LIRA rats (267.3 ± 13.5 mmoL/L x min, *p* < .0001 vs. CON). Dietary restriction also improved glucose tolerance in this model (the postprandial glucose iAUC for RES rats was 241.8 ± 21.4 mmoL/L × min, *p* < .0001 vs. CON). GH treatment alone did not affect glucose tolerance.

Liraglutide treatment, and to a lesser extent food restriction, also shifted the temporal pattern of insulin secretion lower during the OGTT (Figure 3C). Consistent with the results in Figure 2D, fasting insulin concentrations just prior to the OGTT were significantly lower in LIRA (225 ± 24 pmoL/L, mean ± SEM) and GH + LIRA (231 ± 38 pmoL/L) rats, compared with CON rats (409 ± 64 pmoL/L, *p* < .0001 for each comparison, Figure 3C). Despite these differences, there was no discernible effect of treatment on the *amplitude* of the insulin secretion defined by the peak‐nadir, which did not differ between the treatment groups (Figure 3D).

Both groups of liraglutide‐treated rats displayed a significant reduction of fasting GLP‐1 concentrations and/or glucose‐stimulated GLP‐1 secretion (Figures 3E,F). Mean fasting GLP‐1 concentrations were 35% lower in GH + LIRA rats than CON rats (5.27 ± 0.47 pmoL/L, vs. 8.09 ± 0.61 pmoL/L, *p* < .05), while they were significantly increased by 47% in RES rats (11.89 ± 1.05 pmoL/L, *p* = .0005). There was no significant difference (25% decrease, *p* > .05) in fasting GLP‐1 concentrations between CON and LIRA rats. Lastly, GLP‐1 secretion (from 0 to 60 mins) during the OGTT was significantly reduced in LIRA rats (by 63%, *p* < .0001) in LIRA and by 65% in GH + LIRA rats (*p* < .0001), compared with CON rats. GH treatment alone did not have any significant effects on these parameters.

### 3.4 Liraglutide‐treated rats had lower plasma triglyceride levels and liver triglyceride content

**FIGURE 4:**
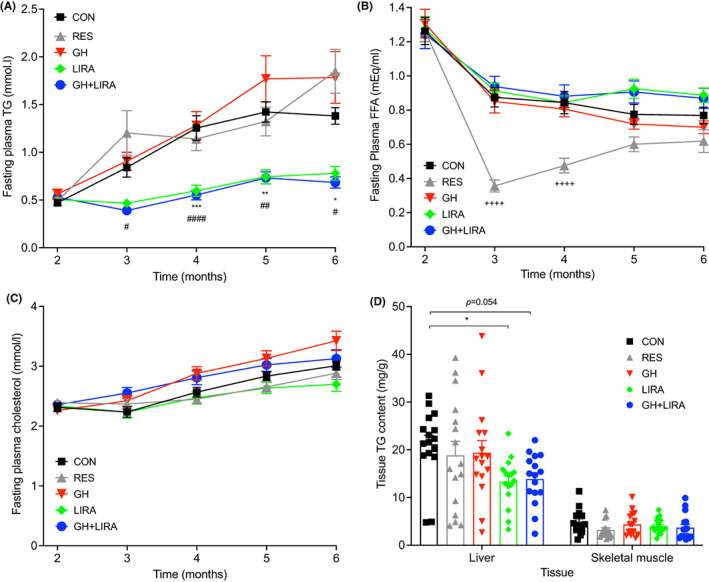
Liraglutide treatment, with or without GH supplementation, protected UCD‐T2DM rats against dyslipidaemia and ectopic lipid accumulation. CON, control; GH, growth hormone; GH + LIRA, growth hormone + liraglutide; LIRA, liraglutide; RES, food‐restricted; *n* = 16/group. Symbols indicate the following significant comparisons: *p < .05, **p < .01, and ***p < .001 for CON versus LIRA; ^#^ *p* < .05, ^##^ *p* < .01, and ^####^ *p* < .0001 for CON versus GH + LIRA; ^++++^ *p* < .0001 for CON versus RES. (A) Fasting triglycerides. Results are shown as mean ± SEM. (B) Fasting concentrations of non‐esterified fatty acids (NEFA). (C) Fasting plasma total cholesterol. (D) Triglyceride content in liver and skeletal muscle; results are shown as scatterplots with mean ± SEM indicated in columns.

Insulin resistance is characterized by impaired suppression of lipolysis in adipose tissue and lipid accumulation in liver and skeletal muscle.^24^ We observed that both groups of liraglutide‐treated rats were protected against dyslipidaemia and ectopic lipid accumulation, while GH treatment alone had no significant effects. After one month of treatment (3 months of age), LIRA and GH‐LIRA rats had significantly lower plasma TG concentrations (Figure 4A) than CON rats, while circulating TG levels in RES and GH rats were unchanged. Liraglutide treatment did not affect fasting FFA concentrations (Figure 4B), although they were markedly reduced by dietary restriction in the first two months of treatment (RES vs. CON, *p* < .0001). Fasting plasma cholesterol concentrations were not affected by any treatment (Figure 4C).

At sacrifice, liver TG content was significantly reduced in LIRA rats (by 37%, *p* < .05 vs. CON rats, Figure 4D), and the 35% decrease in GH + LIRA rats, compared with CON rats approached statistical significance (*p* = .054). Neither food restriction nor GH treatment significantly reduced liver TG content (decreases of 11% and 8%, respectively, *p* > .05). Skeletal muscle TG content did not differ significantly between the treatment groups (Figure 4D). Therefore, consistent with its protection against insulin resistance, liraglutide treatment prevented increases both in circulating TG levels and hepatic TG accumulation in aging UCD‐T2DM rats.

### 3.5 Islet morphology in liraglutide‐treated rats

**FIGURE 5:**
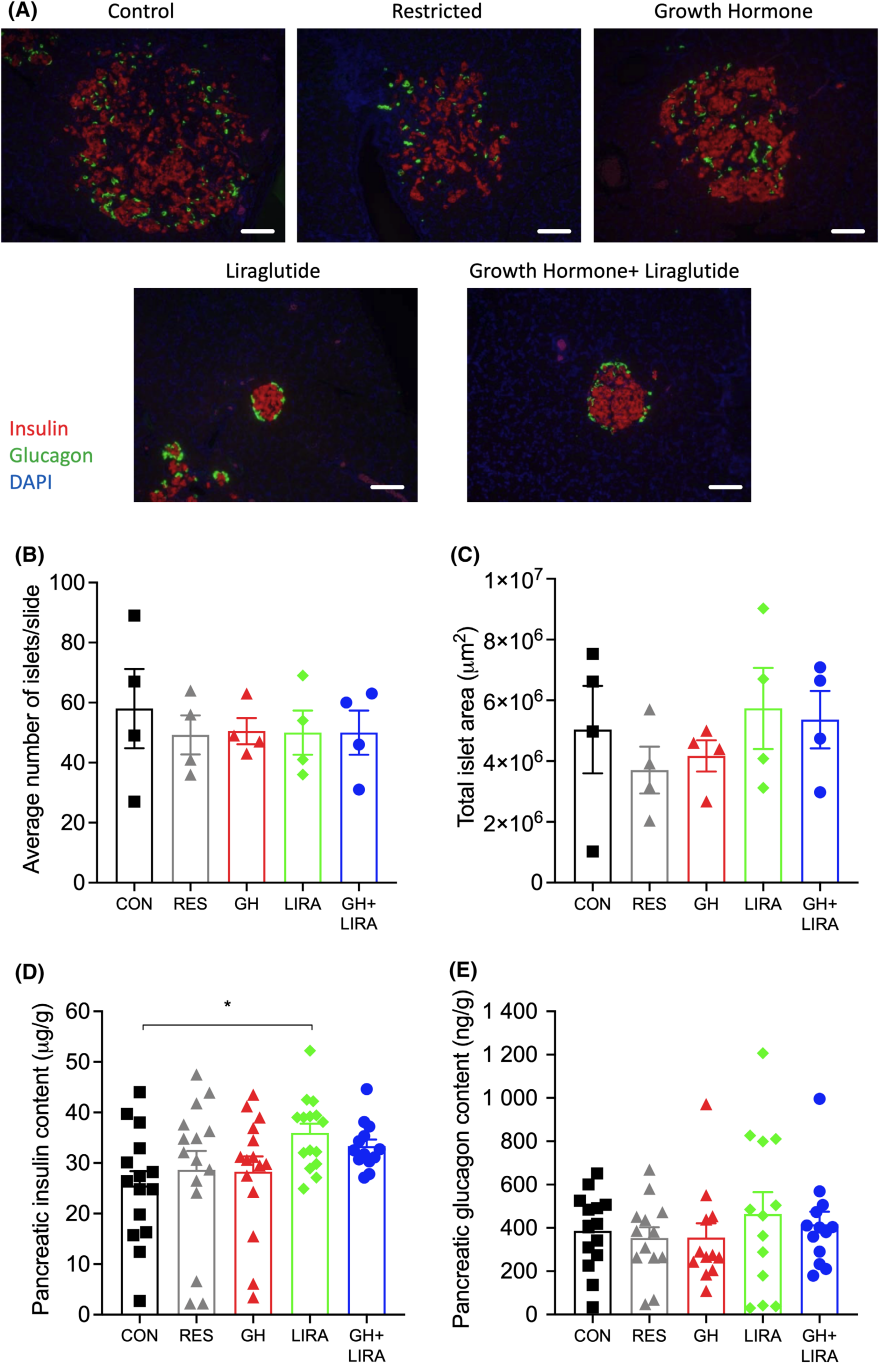
Effects of liraglutide and GH (and their combined effects) on islet morphology, and insulin/glucagon content. CON, control; GH, growth hormone; GH + LIRA, growth hormone + liraglutide; LIRA, liraglutide; RES, food‐restricted. (A) Representative islet images from each group of rats, scale bar = 50 μm; (B) Average number of islets/slide and (C) total islet area (*n* = 4 per group). Results are shown as scatterplots with mean ± SEM indicated in columns. Differences between groups were assessed using one‐way ANOVA, with *p*‐values corrected using Dunnett's multiple comparisons test. (D) Pancreatic insulin content (*n* = 14–15 per group) and (E) pancreatic glucagon content (*n* = 12–14 per group). *p < .05 for CON versus LIRA.

In general, islets from liraglutide‐treated rats had stronger, more dense insulin staining, in comparison to islets from CON, RES and GH rats, in which insulin staining was more diffuse (Figure 5A). The average number of islets per slide (Figure 5B) and the total islet area (Figure 5C) did not differ significantly between groups. Consistent with the increased intensity of insulin staining, pancreatic insulin content was 41% higher in LIRA rats (*p* < .05 vs. CON) and tended to be increased in GH + LIRA rats (30% increase, *p* = .17 vs. CON). Pancreatic glucagon content did not differ between treatment groups (Figure 5E). Therefore, liraglutide treatment appears to have beneficial effects on pancreatic islet morphology and insulin content that are consistent with protection against the development of overt diabetes, while GH treatment did not produce any added beneficial effects on these parameters.

## 4 DISCUSSION

In this study, we examined whether the anti‐diabetic effects of liraglutide would be augmented by GH supplementation in the UCD‐T2DM rat model of type‐2 diabetes. Liraglutide, a medium‐acting GLP‐1 receptor agonist, suppresses food intake by delaying gastric emptying and by activating anorectic pathways in the hypothalamus.^25^ We have previously reported that liraglutide delays the onset of diabetes in our UCD‐T2DM rat model,^16^ and this has been replicated in the OLETF Rat model.^26^, ^27^ In other studies, GH has been reported to exert several beneficial effects on pancreatic β‐cells/islets, including increasing insulin synthesis,^28^, ^29^ GSIS^30^, ^31^ and β‐cell replication^32^ all of which would be considered anti‐diabetic actions. Skeletal muscle mass typically exhibits an inverse relationship with insulin resistance, an important defect underlying the pathophysiology of T2DM^33^, ^34^; and GH treatment increases muscle mass in rats, by increasing cell proliferation and muscle fibre growth.^35^, ^36^ Despite these reports, however, we did not find that GH treatment (0.3 mg/kg daily) provided any additional protection against T2DM in UCD‐T2DM rats when co‐administered with liraglutide, although other physiological effects were observed (see below).

As previously,^16^ and in other comparable studies in rats,^26^, ^37^, ^38^, ^39^ liraglutide attenuated weight gain by reducing food intake. In humans, similarly, weight loss following GLP‐1 agonist treatment is primarily due to reduced food intake.^40^ Here, we found that reduced food intake resulted in lower body weights from the second week of treatment onwards; and after 1 month, insulin sensitivity (QUICKI) was improved, and fasting insulin, leptin and TG concentrations were all significantly reduced, compared to controls. After 1.5 months of treatment, glucose tolerance was improved in liraglutide‐treated rats; and at 3 months, control rats started to develop overt T2DM, while none of the liraglutide‐treated rats developed diabetes during the 4‐month intervention period (up to ~6 months of age).

These effects of liraglutide on T2DM incidence and other obesity‐ or insulin resistance‐related parameters were not further augmented by GH supplementation. T2DM incidence in GH and GH + LIRA rats was not different from CON and LIRA rats, respectively, and fasting insulin concentrations and glucose tolerance were not affected by GH treatment. There were no obvious effects of GH treatment on basal lipolysis, with fasting FFA concentrations being indistinguishable from CON rats (Figure 4B). In other studies, comparable doses of GH have been reported to improve glucose tolerance and reduce hepatic TG content in high‐fat diet (HFD)‐fed mice.^41^ While we considered that the GH dose was insufficient to produce metabolic effects, we did observe a statistically non‐significant (~4%) increase of body weight in GH‐treated rats (Figure 1A), compared with CON rats, without any differences in adiposity. The case for a GH‐mediated increase in lean mass is strengthened by our finding that gastrocnemius muscle mass tended to be higher in GH‐treated rats (by ~6%, *p* = .069 vs. CON rats). This degree of increase is comparable to that observed in transgenic mice overexpressing bovine GH,^42^ which have a ~50‐fold increase of circulating GH levels.^43^ In the present study, we were not able to determine GH concentrations in GH‐treated rats, as the morning GH dose was administered *after* the collection of fasting blood samples. In normal rats, the half‐life of exogenous GH is <18 minutes.^44^ Interestingly, GH treatment also tended to lower fasting plasma leptin concentrations in our model (Figure 1E), consistent with its reported effects in GH‐deficient humans.^45^

We concede that the GH dose used here (0.3 mg/kg) may have been insufficient to produce any direct effects on β‐cells/islets. Most of the studies on GH's pancreatic effects have been performed in isolated pancreata or islets in vitro. However, the most plausible conclusion is that GH did not produce detectable effects that were independent of the potent effects of liraglutide. It is likely that other experimental conditions (e.g., prolonged exposure to HFD) may be required to detect the anti‐diabetic effects of GH.

Finally, the current study also clarified some of the mechanisms underlying the anti‐diabetic effects of liraglutide. Liraglutide is known to improve T2DM by preventing accumulation of toxic fatty acid metabolites in tissues such as skeletal muscle, liver, adipocytes and β‐cells that contributes to insulin resistance and β‐cell dysfunction, respectively, in type 2 diabetes.^46^ In UCD‐T2DM rats, the clearest evidence for this effect can be assessed from fasting plasma triglyceride concentrations (Figure 4A) and hepatic TG content (Figure 4D), both of which were significantly reduced in LIRA and GH + LIRA rats, compared with CON rats. Insulin resistance in this model is more likely to result from hepatic insulin resistance (rather than in skeletal muscle), considering that there were no differences in skeletal muscle TG content between groups (Figure 4D) as we previously reported.^16^ In clinical studies, administration of GLP‐1 agonists improved hepatic insulin sensitivity and reduced hepatic de novo lipogenesis in humans,^10^ a finding which has also been observed in fructose‐fed hamsters.^47^ It is also possible that the major anti‐diabetic effect of liraglutide is to improve β‐cell function (as shown in recent human studies)^48^, ^49^: in the present study, rats that eventually developed diabetes (5 CON rats, 3 GH rats and 1 RES rat, but no liraglutide‐treated rats) exhibited fasting hyperinsulinaemia prior to developing overt diabetes. However, this phenotype was not explored further, due to limited statistical power. Adiponectin, an insulin‐sensitizing adipokine, promotes fatty acid oxidation and suppresses gluconeogenesis in the liver,^22^ by regulating the expression of rate‐limiting genes.^50^ In the present study, however, the improved glucose and lipid metabolism in liraglutide‐treated rats could not be attributed to changes in adiponectin concentrations (Figure 1F).

Interestingly, GH also protects against fatty acid‐induced damage in β‐cells^51^; although in rats, continuous GH treatment (5 μg/h; 7 days) has been reported to increase the incorporation of [1‐14C] oleic acid into hepatic triglycerides.^52^ Regardless, our results indicate that the beneficial effects of extended liraglutide treatment on hepatic TG content exceeded any potential detrimental effects of GH in this model.

In addition to the novel hypothesis tested, a further strength of the study was the addition of the group of weight‐matched food‐restricted (RES) rats, which revealed that liraglutide had additional metabolic benefits on parameters related to T2DM beyond restriction of food intake and reduction of body weight. *Post hoc* analyses revealed that these benefits included significant reductions of visceral (mesenteric and retroperitoneal) adipose depot weights, fasting insulin concentrations (from 3 to 6 months) and an index of insulin resistance (QUICKI), as well as lower fasting plasma TG and increases of pancreatic islet area and insulin content.

Interestingly, energy expenditure was significantly increased in LIRA and GH + LIRA rats compared with RES rats (Figure 1C,D). This is consistent with our previous liraglutide study,^16^ in which liraglutide‐treated rats consumed more food than RES rats to maintain the same body weight. One limitation of the calorimetry results, however, was that energy expenditure was not normalized for lean mass; therefore, these potential effects of liraglutide on energy expenditure could be confounded by differences in body composition. In the present study, however, differences in lean mass between RES and liraglutide‐treated rats were unlikely, based on their similar gastrocnemius muscle and liver weights (Table 1). Another limitation of the present study is that a longer duration of treatment may be necessary to fully assess the effects of liraglutide and GH when administered together on pancreatic islet morphology and the many other metabolic parameters we assessed.

While our results are consistent with a previous report of hepatic AMPK activation by liraglutide,^53^ leading to suppression of hepatic lipogenesis and a subsequent reduction in circulating TG concentrations and hepatic TG content as a major mechanism for its beneficial metabolic effects, there are other pathways, such as changes of hepatic fatty acid oxidation and/or the accumulation of intermediate lipid species (e.g., DAGs, ceramides) that antagonize insulin action, that have yet to be thoroughly investigated.

In conclusion, chronic administration of GH to liraglutide‐treated UCD‐T2DM rats did not further delay the onset of T2DM and did not significantly alter other related metabolic or endocrine outcomes (including pancreatic islet area or insulin content) in this model.

## AUTHOR CONTRIBUTIONS

**Michael Swarbrick:** Data curation (equal); formal analysis (lead); methodology (equal); project administration (equal); visualization (equal); writing – original draft (lead). **Chad Cox:** Data curation (equal); investigation (equal); writing – review and editing (supporting). **James Graham:** Data curation (equal); investigation (equal); methodology (equal); project administration (equal); writing – review and editing (equal). **Lotte Knudsen:** Conceptualization (equal); funding acquisition (equal); resources (equal); writing – review and editing (supporting). **Kimber Stanhope:** Investigation (supporting); methodology (supporting); project administration (supporting); writing – review and editing (supporting). **Kirsten Raun:** Conceptualization (equal); funding acquisition (equal); methodology (equal); resources (equal); writing – review and editing (supporting). **Peter Havel:** Conceptualization (lead); funding acquisition (lead); methodology (equal); project administration (equal); resources (equal); writing – review and editing (equal).

## CONFLICT OF INTEREST

LBK and KR are full‐time employees of Novo Nordisk and are also minor stock owners as part of an employee offering program. Novo Nordisk markets liraglutide for treatment of type 2 diabetes. PJH's laboratory received funding from Novo Nordisk to conduct this project. The remaining authors have no relevant conflicts of interest.

## Supporting information


Figure S1.

## Data Availability

The data that support the findings of this study are available from the corresponding author upon reasonable request.
