# Supplementary material for: Growth hormone treatment does not augment the anti‐diabetic effects of liraglutide in UCD‐T2DM rats
Source: Endocrinol Diabetes Metab. 2022 Dec 8;6(1):e392. doi: 10.1002/edm2.392 (PMC9836246; doi:10.1002/edm2.392)
Supplement: Supplementary file 1 — Figure S1. [file EDM2-6-e392-s001.docx]

**Supplementary Figure 1** for Swarbrick MM et al. “Growth Hormone Treatment does not Augment the Anti-Diabetic Effects of Liraglutide”. Submitted to *Endocrinology, Diabetes and Metabolism*.


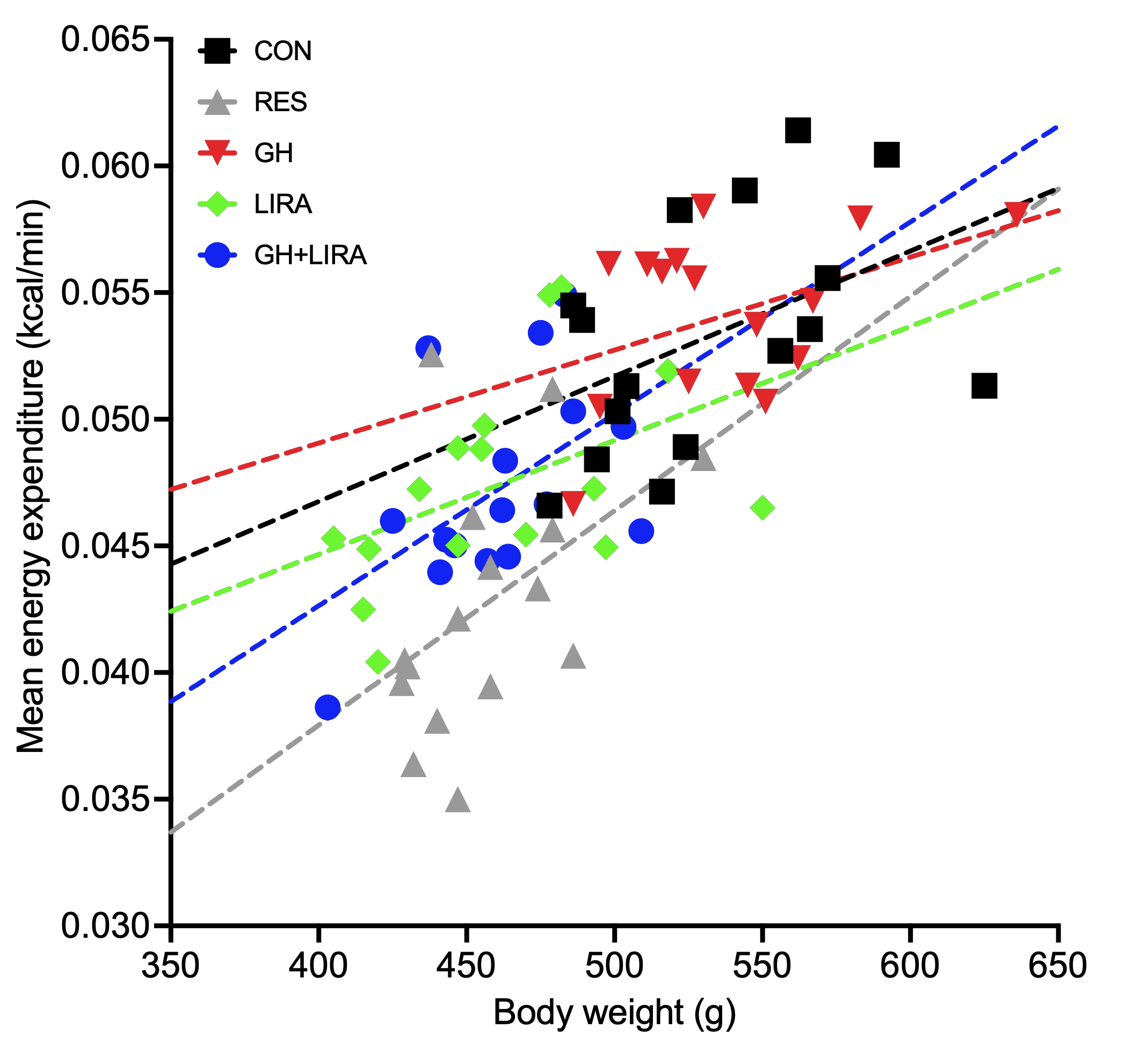


**Legend:** Mean energy expenditure and body weight were plotted for all 5 groups of rats (CON, RES, GH, LIRA, GH+LIRA, n=16/group). Differences in the slope of the lines were tested using linear regression (GraphPad Prism v9 for macOS, San Diego, CA, U.S.A.).

**Results:**

- There were no significant effects of GH treatment on energy expenditure, when adjusted for differences in body weight: for CON vs GH, *F*_1,28_= 0.1379, *P*=0.71
- There were no significant effects of liraglutide treatment on energy expenditure, when adjusted for differences in body weight: for CON vs. LIRA, *F*_1,28_= 0.01558, *P*=0.90
- There were no significant effects of GH and liraglutide in combination on energy expenditure, when adjusted for body weight: for CON vs GH+LIRA, *F*_1,28_= 0.3556, *P*=0.56
- There were no significant effects of energy restriction on energy expenditure, when adjusted for body weight: for CON vs RES, *F*_1,28_= 0.4945, *P*=0.49
- Any differences in energy expenditure between RES and liraglutide-treated rats were not significant when body weight was taken into account: for RES vs LIRA, *F*_1,28_= 0.6805, *P*=0.42; for RES vs GH+LIRA, *F*_1,28_= 0.02600, *P*=0.87.
